# Downregulation of adaptor protein MyD88 compromises the angiogenic potential of B16 murine melanoma

**DOI:** 10.1371/journal.pone.0179897

**Published:** 2017-06-29

**Authors:** Lucas Daniel Trucco, Emiliano Roselli, Paula Araya, Nicolás Gonzalo Nuñez, Hebe Agustina Mena, José Luis Bocco, Soledad Negrotto, Mariana Maccioni

**Affiliations:** 1 Centro de Investigaciones en Bioquímica Clínica e Inmunología (CIBICI), Consejo Nacional de Investigaciones Científicas y Técnicas (CONICET), Departamento de Bioquímica Clínica, Facultad de Ciencias Químicas, Universidad Nacional de Córdoba, Córdoba, Argentina; 2 Laboratoire de Transfert, INSERM U932, Laboratoire d'Immunologie, Institute Curie, París, France; 3 Laboratorio de Trombosis Experimental, Academia Nacional de Medicina, Buenos Aires, Argentina; Medical College of Wisconsin, UNITED STATES

## Abstract

The mechanisms that link inflammatory responses to cancer development remain a subject of intense investigation, emphasizing the need to better understand the cellular and molecular pathways that create a tumor promoting microenvironment. The myeloid differentiation primary response protein MyD88 acts as a main adaptor molecule for the signaling cascades initiated from Toll-like receptors (TLRs) and the interleukin 1 receptor (IL-1R). MyD88 has been shown to contribute to tumorigenesis in many inflammation-associated cancer models. In this study, we sought to better define the role of MyD88 in neoplastic cells using a murine melanoma model. Herein, we have demonstrated that MyD88 expression is required to maintain the angiogenic switch that supports B16 melanoma growth. By knocking down MyD88 we reduced TLR-mediated NF-κB activation with no evident effects over cell proliferation and survival. In addition, MyD88 downregulation was associated with a decrease of HIF1α levels and its target gene VEGF, in correlation with an impaired capability to induce capillary sprouting and tube formation of endothelial cells. Melanomas developed from cells lacking MyD88 showed an enhanced secretion of chemoattractant ligands such as CCL2, CXCL10 and CXCL1 and have an improved infiltration of macrophages to the tumor site. Our results imply that cell-autonomous signaling through MyD88 is required to sustain tumor growth and underscore its function as an important positive modulator of tumor angiogenesis.

## Introduction

The myeloid differentiation primary response protein MyD88 acts as a pivotal adaptor molecule for the signaling pathways initiated from Toll-like receptors (TLRs) and the interleukin 1 receptor (IL-1R). Upon activation by their respective ligands, these receptors engage MyD88 driving the assembly of a multiprotein signaling complex which ultimately triggers the expression of several genes involved in the pro-inflammatory response [1]. Several lines of evidence have suggested that MyD88 plays an important role in tumor development. In addition, high MyD88 expression was described in several tumors [2] and enhanced levels of MyD88 were associated with poor prognosis in hepatocellular carcinoma, colorectal and ovarian cancers [3–5].

Stimulation of TLRs is mediated by pathogen associated molecular patterns including lipopolysaccharide (LPS), lipopeptides, flagellin, bacterial DNA and viral double-stranded RNA. Besides microbial products, TLRs also binds endogenous, host-derived danger associated molecular patterns (DAMPs), i.e., molecules that are released or exposed by stressed or dying cells, for example high mobility group box 1 (HMGB1), heat shock proteins and β-defensins [6]. Thirteen different TLRs have been characterized in mammals and all TLRs family members transmit signals through MyD88 with the exception of TLR3, which uses the adaptor protein TICAM1 (also known as TRIF). Binding of ligands leads to the dimerization of TLRs and the recruitment of MyD88, which in turn engages different signaling molecules resulting in the activation of the mitogen-activated protein kinases (MAPKs), the phosphatidylinositol 3-kinase (PI3K)/AKT pathway and the transcription factor NF-κB (p50/p65). Subsequently, these cascades induce the expression of key inflammatory cytokines such as tumor necrosis factor (TNF)-α and interleukins IL-1β, IL-6 and IL-8 [7].

Previous reports have shown that MyD88 contributes to tumorigenesis in many inflammation-associated cancer models. Ablation of MyD88 impairs intestinal tumor formation induced by the administration of the carcinogen azoxymethane and the spontaneous tumor development in mice with heterozygous mutation in the adenomatous polyposis coli gene, as well as diethylnitrosamine-induced hepatocarcinogenesis associated with a reduced IL-6 production [8,9]. Additionally, MyD88 is an essential positive regulator for chemically induced skin papilloma and fibrosarcoma formation [10]. In contrast, a protective role of MyD88 was described for chronic colitis-associated colon cancer formation and gastric cancer development following infection with *Helicobacter* [11,12]. Moreover, non-inflammatory functions exerted by MyD88 regulate the proliferation, metastasis and chemoresistance of malignant cells. For instance, it was found that MyD88 is required for oncogenic Ras-mediated transformation in fibroblast and keratinocytes, having the IL-1R/MyD88/NF-κB axis a mandatory role in the latter [13,14]. Also, the TLR4/MyD88 signaling is implicated in the acquisition of paclitaxel resistance in ovarian cancer and MyD88 inhibition renders colon cancer cell lines susceptible to apoptosis mediated by cisplatin [15,16]. However, the precise mechanisms whereby MyD88 act intrinsically in tumor cells to sustain cancer progression remain poorly understood.

A growing capillary network is strictly necessary for ensuring sufficient supply of oxygen and nutrients in expanding solid tumors. The process of formation of new blood vessels via sprouting or splitting of the pre-existing vasculature, termed angiogenesis, is initiated and controlled by multiple growth factors produced by the cancer cells and the cells from the stromal microenvironment [17]. Among the pro-angiogenic factors, the vascular endothelial growth factor (VEGF) is especially important. VEGF is a potent promoter of cell survival, growth and migration of endothelial cells and a key mediator in the angiogenic switch from the avascular to vascular phenotype mainly via the VEGFR2 receptor [18]. The hypoxic tumor milieu induces the upregulation of VEGF gene expression, mediated by the activation of the hypoxia-inducible factor 1α (HIF1α), creating a VEGF gradient that provides chemotactic signals for the sprouting of vascular cells [19]. Stimuli other than oxygen deprivation can also induce HIF1α activity in tumor tissues leading to increases in VEGF in normoxic conditions including oncogenic signaling and loss-of-function mutations in tumor-suppressor genes like *p53* and *Pten* [20]. Nevertheless, there are other angiogenic factors with documented effects on tumor vessel growth such as members of the fibroblast growth factor (FGF), platelet-derived growth factor, angiopoietin, and transforming growth factor-β families, IL-8 or the VEGF homolog placenta growth factor (PlGF) [21].

In this study, we sought to better define the role of MyD88 in neoplastic cells using a murine melanoma model. We evaluate how MyD88 downregulation influences the proliferation, cell cycle and survival of B16 cells, and its ability to develop tumors when grafted into syngeneic mice. Our results imply that cell-autonomous signaling through MyD88 is required to sustain tumor growth and underscore its function as an important positive modulator for tumor angiogenesis.

## Results

### Characterization of MyD88-silenced B16 melanoma cells

**Fig 1:**
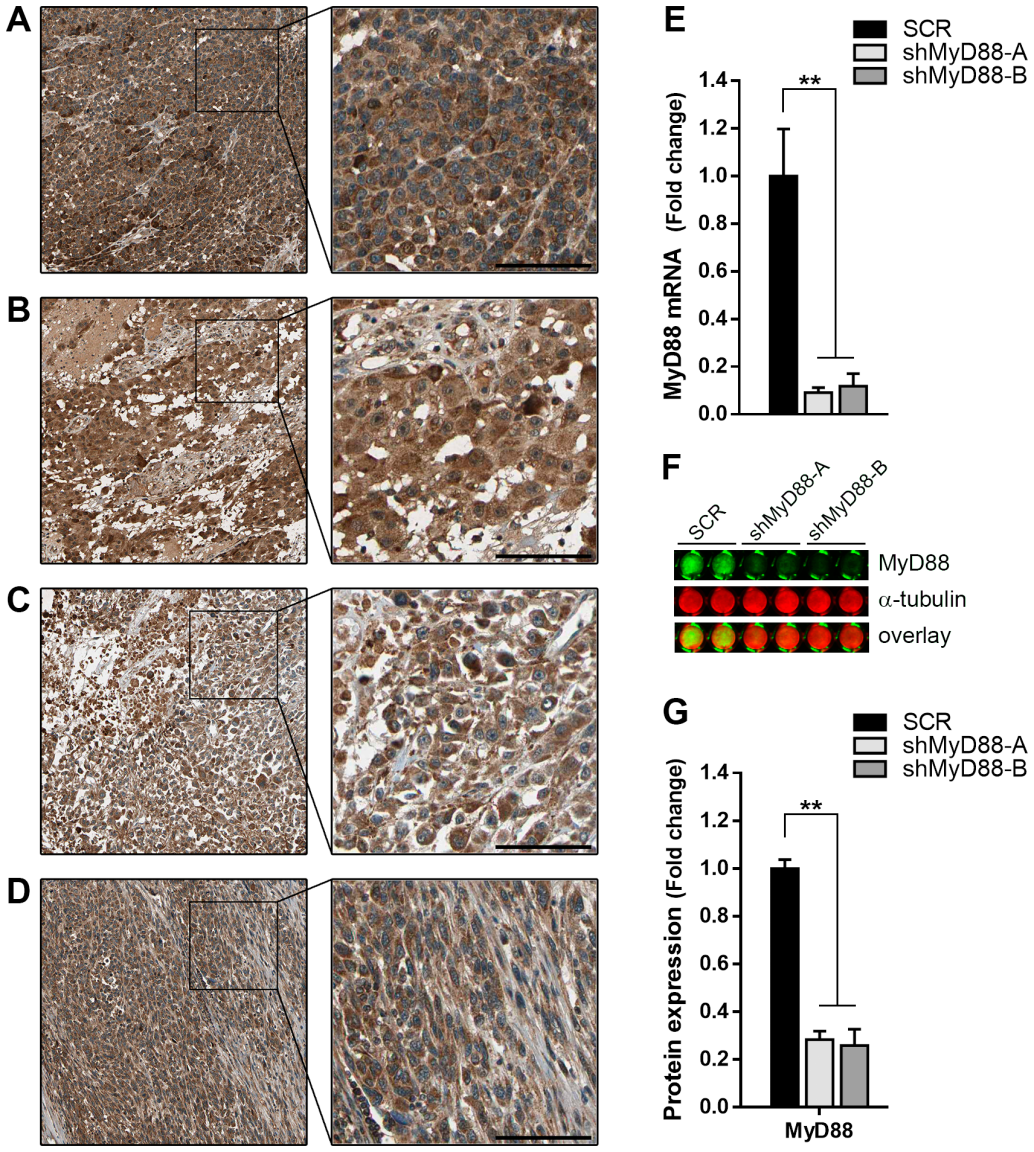
Stable downregulation of MyD88 in murine melanoma cells. **(A-B)** Representative immunohistochemistry images of tissue samples from primary and **(C-D)** metastatic human melanomas stained with antiMyD88 antibody (brown). The nuclei were counter-stained with Hematoxylin (blue). All images were obtained from The Human Protein Atlas; http://www.proteinatlas.org/ENSG00000172936-MYD88/cancer. Scale bar = 100μμm. **(E)** Quantitative RT-PCR analysis of MyD88 mRNA expression in B16 cells stably transduced with lentiviruses to express either scrambled (SCR) or MyD88-specific shRNAs (shMyD88-A and -B). Data represent fold increase ± SD after normalization with GAPDH mRNA (** p<0.001). **(F)** Protein expression of MyD88 was determined by In-Cell Western analysis. Representative output image showing whole-cell fluorescence from duplicate wells of a 96-well plate captured with an Odyssey infrared scanner. α-tubulin signal was used for normalization. **(G)** Graph represents the fold change in normalized signal intensity values respect to SCR cells. Data are shown as means ± SEM (** p<0.001; * p<0.05).

According to data available from The Human Protein Atlas, MyD88 protein is highly overexpressed in primary and metastatic cutaneous melanoma (Fig 1A–1D), as well as in melanoma derived cell lines (http://www.proteinatlas.org) [22]. This evidence suggests that aberrant elevation of MyD88 expression may have a critical role in maintaining the malignant features in this type of cancer. To investigate the impact of MyD88 intrinsical activity in tumor cells, we used short hairpin RNA (shRNA) lentiviral vectors to reduce endogenous MyD88 levels in B16 melanoma cells. The B16 cells stably transduced with two independent shRNAs against MyD88 (shMyD88-A and shMyD88-B) showed a strong suppression of MyD88 expression, with >90% and >75% decrease in mRNA and protein levels, respectively, compared with cells transduced with a scrambled shRNA control (SCR) (Fig 1E–1G).

**Fig 2:**
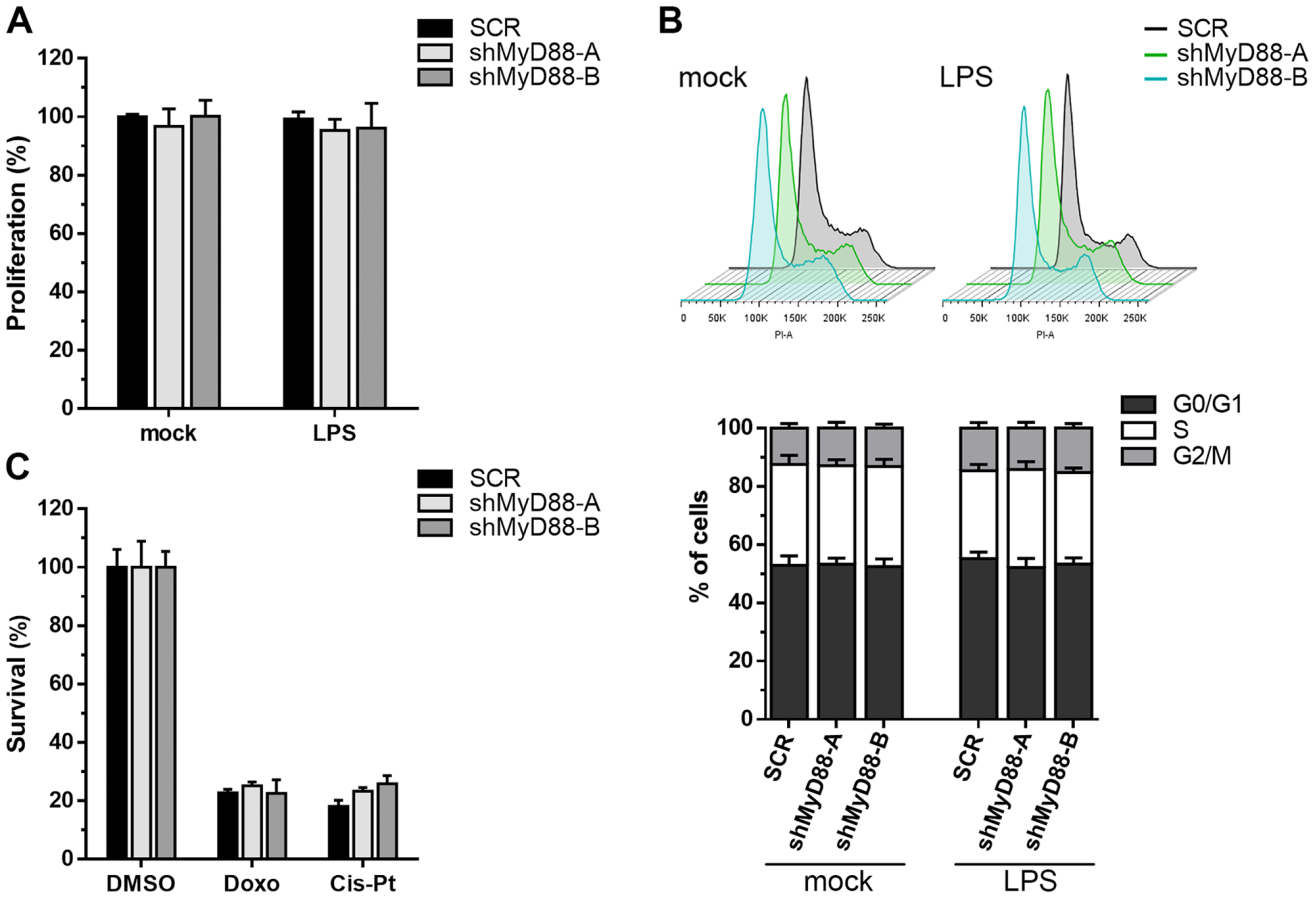
Knockdown of MyD88 did not compromise B16 cell growth. Experiments were performed in B16 cells expressing either SCR or MyD88-specific shRNAs and incubated in the absence (mock) or presence of LPS (1 μg/mL) as indicated. **(A)** Cell proliferation evaluated in cells cultured at 10% serum for 48 h. The data are presented as percentage over SCR control cells ± SD. **(B)** Cell cycle distribution of B16 cells in exponential growth phase was analyzed by flow cytometry. Cell cycle profiles (top) and percentages of cells in G0/G1, S, and G2/M phases (bottom) are shown. **(C)** Cell viability of B16 cells exposed to doxorubicin (Doxo; 1 μM) or cisplatin (Cis-Pt; 20 μμg/ml) for 48 h was determined using an MTS assay. Survival of cells treated with vehicle (DMSO) was established as 100%.

To evaluate the possible contribution of MyD88 in the regulation of cellular proliferation, we compared the growth rate and cell cycle distribution of shMyD88 and SCR B16 cells *in vitro*. In order to study the effect of the TLR/MyD88 signaling, cells were stimulated with LPS, an integral glycolipid found in the cell wall of Gram-negative bacteria which is recognized and activates TLR4 [23]. As shown in Fig 2A, no differences in the cell proliferation rates of MyD88-knockdown and SCR cells were observed, neither in basal conditions nor under LPS treatment. Accordingly, when the cell cycle distribution was analyzed in the three cell lines, we found equivalent percentages of cells in the different phases of the cell cycle and no effect of LPS stimulation (Fig 2B). We also tested whether MyD88 may alter the sensitivity to apoptosis mediated by genotoxic drugs. Cell survival assays performed for MyD88-knockdown and SCR B16 cells exposed to doxorubicin or cisplatin demonstrated that MyD88 depletion had no influence in the cell death induced by these chemotherapeutic agents (Fig 2C). Together, these results indicate that MyD88 downregulation do not impact significantly the *in vitro* growth capacity and apoptotic response of B16 cells.

### MyD88 signaling regulates NF-κB activity and HIF1α expression

**Fig 3:**
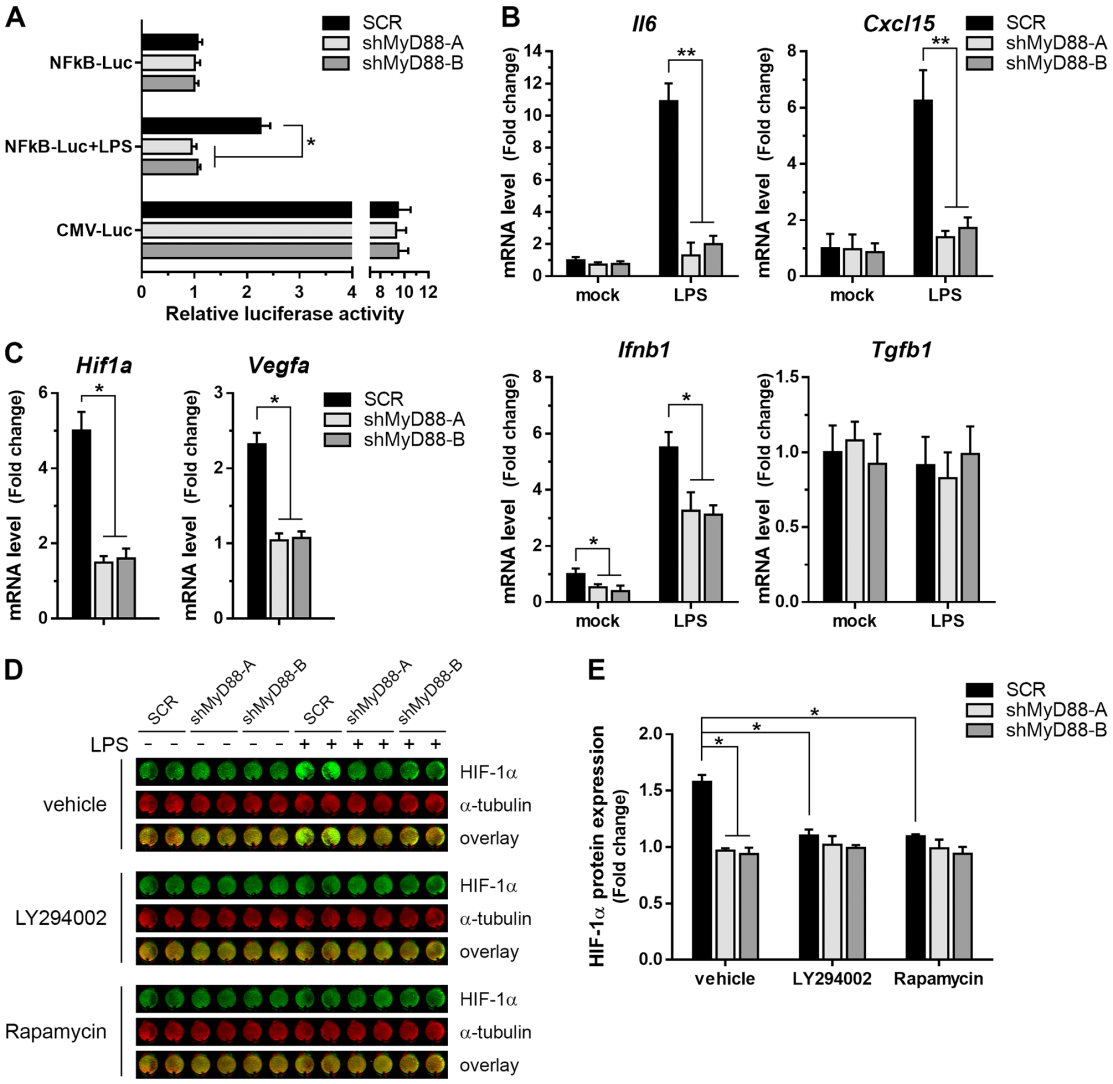
Reduced NF-κB activity and HIF1α expression in MyD88-silenced cells. **(A)** NF-κB transcriptional activity. Following co-transfection of SCR and shMyD88 cells with a NF-κB-driven luciferase reporter (NFkB-Luc) along with the normalizing vector coding for renilla, cells were stimulated with LPS (1 μg/mL) or left untreated. Luciferase activity was measured in cell extracts obtained 24 h later. A plasmid with a cytomegalovirus promoter (CMV-Luc) was used to control for equal transcription rate between cell lines. Transcriptional activity is expressed as fold increase ± SD, with the activity from unstimulated SCR cells arbitrarily set at 1 (* p<0.01). **(B)** Quantitative RT-PCR analysis of IL6, CXCL15, IFN-β, and TGF-β mRNA expression in SCR and shMyD88 cells treated or not with LPS. Data represent fold change ± SD relative to untreated SCR cells after normalization with GAPDH expression (** p<0.001; * p<0.05). **(C)** Levels of HIF1α and VEGFA gene expression after LPS stimulation measured by qRT-PCR. Fold induction ± SD respect to the values from each unstimulated cell line were calculated after normalization with GAPDH mRNA (* p<0.01). **(D)** Protein expression of HIF1α and α-tubulin (loading control) determined by In-Cell Western. B16 SCR and shMyD88 cells were pretreated with vehicle (DMSO), LY294002 (PI3K inhibitor), or Rapamycin (mTOR inhibitor) for 20 min and incubated in the absence or presence of LPS for 18 h prior to analysis. Representative image from wells seeded in duplicates obtained with an Odyssey infrared scanner. **(E)** Quantification of HIF1α protein levels in LPS treated cells. Values were normalized to α-tubulin signal and presented as fold change ± SEM relative to those of the non-LPS-treated cells (* p<0.01).

The transcription factor NF-κB constitute a master regulator of the responses mediated by MyD88-dependent pathways. Its activation involves the phosphorylation of IκBα, an NF-κB inhibitor; which enables IκBα ubiquitination and proteasome-mediated degradation, leading to increased translocation of NF-κB to the nucleus, where NF-κB binds to target genes promoter regions to activate transcription [24]. Thus, to study the effect of MyD88 depletion on NF-κB activation we measured reporter gene activity using an NF-κB reporter plasmid. While all three B16 cell lines showed comparable basal NF-κB reporter activities, MyD88 silenced cells fail to respond to TLR4 stimulation with LPS compared with SCR cells, in which it was able to induce the activation of the NF-κB reporter gene (Fig 3A). Of note, the activities of a non-related (CMV) promoter were similar between control and shMyD88 B16 cells (Fig 3A). Next, we looked to correlate this result with the expression pattern of inflammation-related genes. Albeit unstimulated cells present no differences in the mRNA levels of IL-6, the strong LPS-mediated upregulation of this cytokine was inhibited in the MyD88-knockdown cells (Fig 3B). A similar profile was observed for the expression of the chemokine CXCL15. Instead, shMyD88 cells displayed a different pattern of expression of the type I interferon IFN-β, having reduced levels compared to SCR cells but able to induce its expression upon LPS treatment (Fig 3B), indicating that TLR4-mediated induction of IFN-β is independent of MyD88. In contrast, the transcript levels of transforming growth factor beta (TGF-β) remained unaffected by MyD88 downregulation.

A synergistic crosstalk between NF-κB and HIF1α has been described, comprising control mechanisms that function under normoxic as well as hypoxic conditions [25,26]. Indeed, HIF1α gene expression and protein accumulation could be induced through NF-κB activation downstream of TLRs [27]. Hence, we evaluated whether LPS modulates HIF1α expression in B16 melanoma cells. Quantification of HIF1α mRNA levels revealed a 5-fold induction upon LPS exposure in SCR cells, over untreated controls (Fig 3C), with a concurrent increase in the abundance of HIF1α protein (Fig 3D). This increment in HIF1α levels correlated with an enhanced expression of VEGF, a direct target gene of HIF transcription factor (Fig 3C). Likewise, no changes in neither VEGF nor HIF1α expression were observed in cells expressing the MyD88 specific shRNAs treated with LPS (Fig 3C and 3E), which suggest that TLR4 activation upregulates HIF1α and VEGF expression in a MyD88-dependent manner. Then, to determine the signaling pathways mediating the TLR-induced rise of HIF1α levels, B16 cell lines were treated with LY294002, rapamycin, or U0126 which are pharmacological inhibitors of PI3K, mammalian target of rapamycin (mTOR) and MAPK-kinase MEK, respectively. Treatment with LY294002 or rapamycin blocked HIF1α induction under LPS stimulation (Fig 3D and 3E), whereas U0126 did not alter HIF1α expression in SCR B16 cells (). Meanwhile, levels of HIF1α were not modified by any of the kinase inhibitors tested in MyD88-knockdown cells. Collectively, these results indicate that PI3K/AKT/mTOR signaling downstream of MyD88 is required for HIF1α expression upon TLR activation.

### MyD88 influences the angiogenic profile in B16 murine melanoma cells

**Fig 4:**
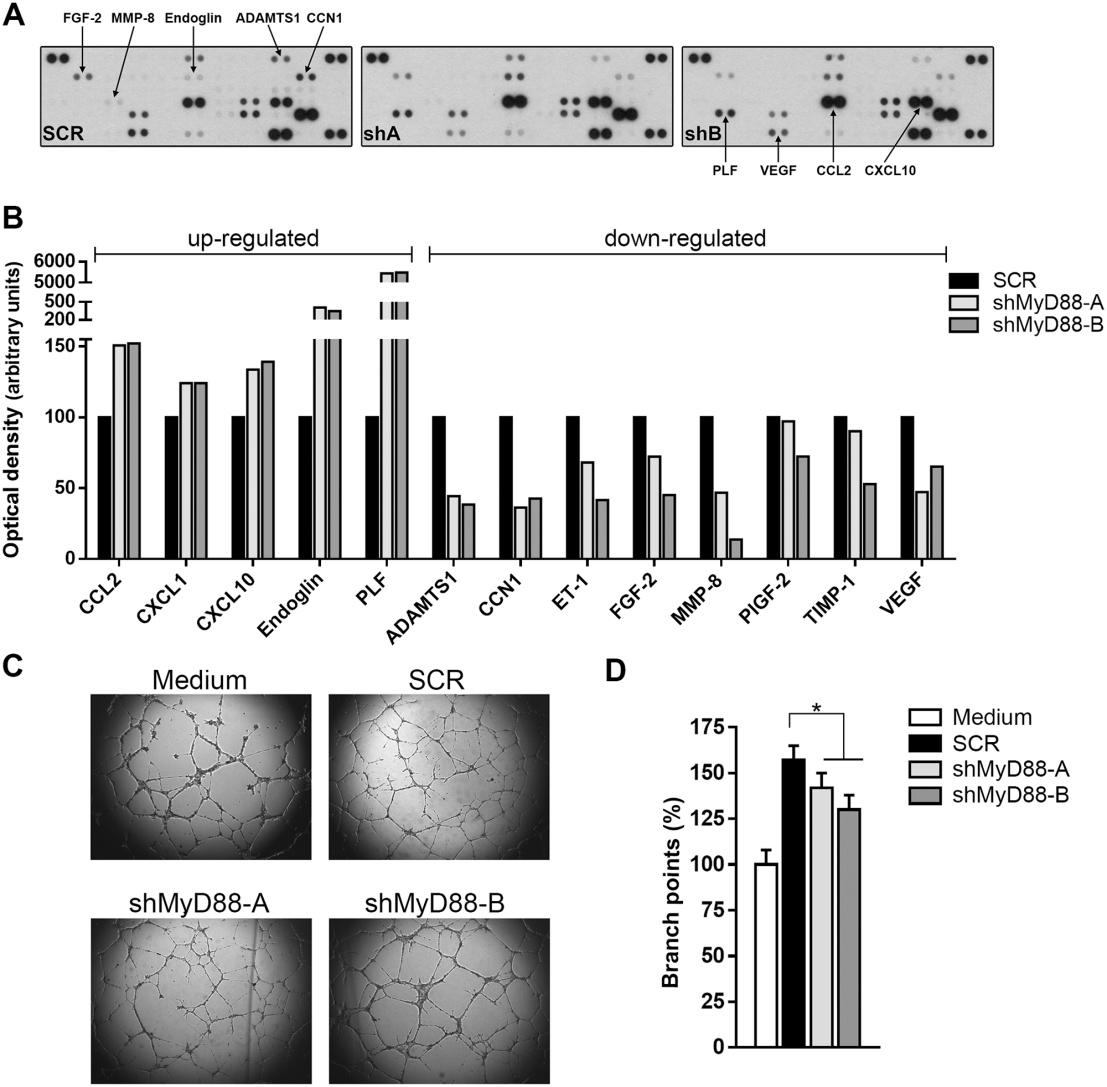
Downregulation of MyD88 alters the secretion of angiogenic mediators. Murine angiogenesis antibody arrays were probed with conditioned media collected from B16 cells. **(A)** Blot images from SCR and shMyD88 (shA and shB) cells. **(B)** Quantitative analysis of array data for down- and up-regulated proteins. Results shown are representative of two independent experiments **(C)** Representative photomicrographs of *in vitro* tube formation assay. Microvascular endothelial cells (HMEC-1) were seeded onto matrigel-coated slides and incubated with culture medium (media) and B16 conditioned media from the indicated cell lines **(D)** Quantitation of HMEC-1 branch points. Values are expressed as percentages ± SEM respect to those obtained with supernatants from SCR cells (* p<0.05).

The results described above suggest that intrinsic MyD88-dependent signaling might influence the ability of tumor cells to promote a pro-angiogenic tissue microenvironment. With the purpose of address this issue, we studied the secreted protein profile in B16 melanoma cells. We subjected conditioned media from SCR or MyD88-downregulated cells to a murine antibody array that detects angiogenesis-related factors (Fig 4A). As shown in Fig 4B, ablation of MyD88 expression leads to a distinctive secretion of many soluble mediators involved in the regulation of tumor vasculature. Notably, shMyD88 cells showed a reduced production of the proangiogenic factors VEGF, FGF-2, PlGF, Endothelin-1 (ET-1), and matricellular protein CCN1 (also known as cysteine-rich angiogenic inducer 61, CYR61), as well as the reduction of the antiangiogenic factor ADAMTS1. Furthermore, decreased levels of metalloproteinase 8 (MMP-8) and its inhibitor TIMP-1 were detected in shMyD88-A and -B cell supernatants. By contrast, MyD88 knockdown significantly enhances the release of the proangiogenic factors endoglin and proliferin (PLF), concurrent with increased secretion of chemokines (C-X-C motif) ligand 1 (CXCL1), CXCL10, and (C-C motif) ligand 2 (CCL2) which are associated with the recruitment or activation of leukocytes.

To ascertain whether factors secreted by B16 cells were involved in tumor angiogenesis, and evaluate whether signaling via MyD88 was required, we explored the influence of conditioned medium from melanoma cells on endothelial cell tube formation. To this end, human microvascular endothelial cells (HMEC-1) were cultured on matrigel in the presence of culture medium or conditioned supernatants from SCR or shMyD88 cells and then examined the next day for tubulogenesis (Fig 4C). Functionally, capillary-like structure formation that mimic blood vessel is one of the most important morphological changes during angiogenesis and can be quantified by counting the sprouting and branching of endothelial cells [28]. The assays revealed that conditioned media from MyD88-silenced cells have a reduced potential to promote tube-formation of HMEC-1, as judged by the number of branch points, compared with SCR control conditioned media (Fig 4D). Overall, these finding indicates that MyD88 expression supports a secretory phenotype that contributes to sustain a proangiogenic tumor microenvironment.

### MyD88 downregulation hampers melanoma growth *in vivo*

**Fig 5:**
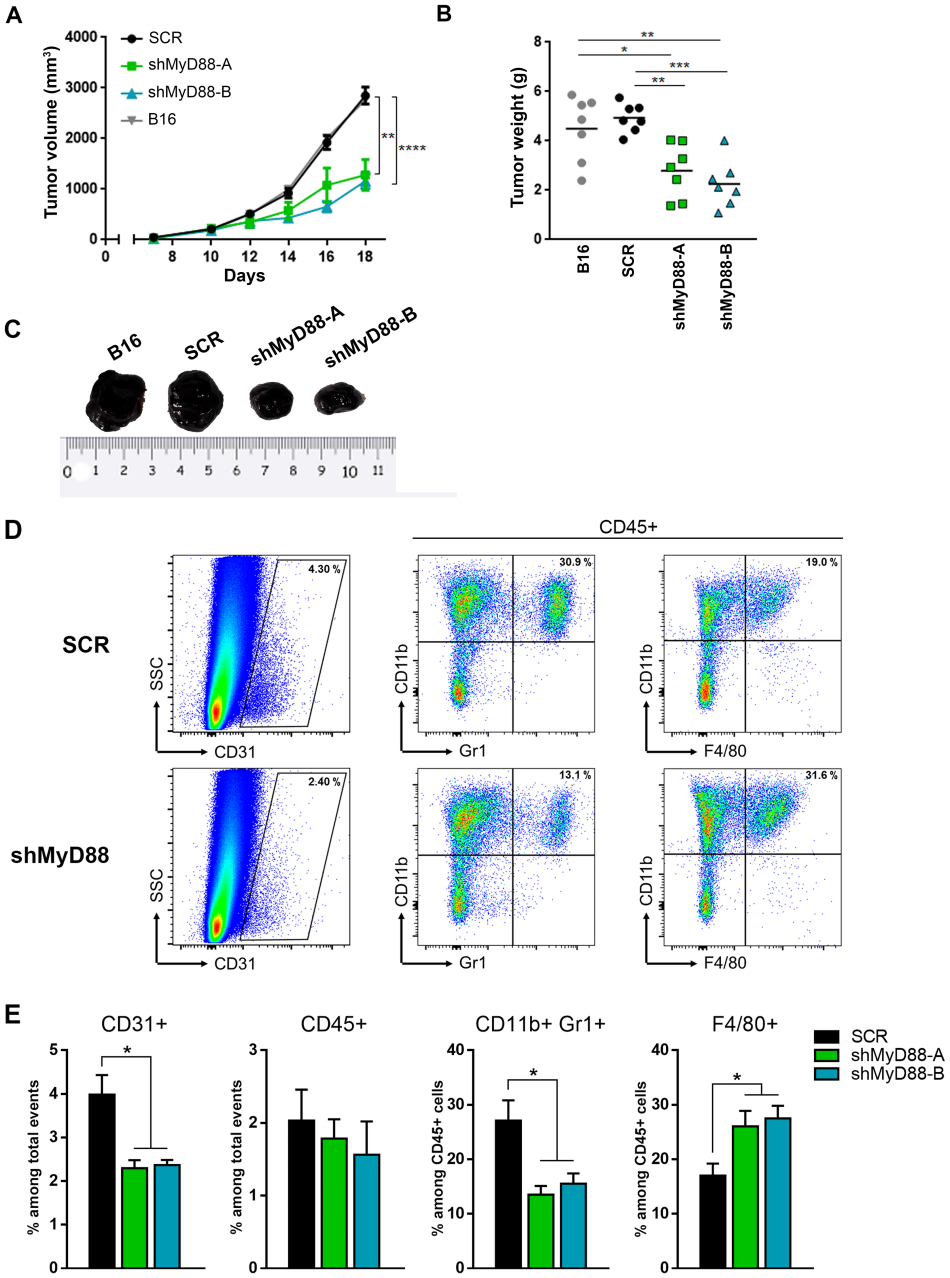
MyD88-knockdown cells showed a decreased tumor growth *in vivo*. Non-transduced B16 cells and B16 cells expressing SCR or MyD88-specific shRNAs were injected subcutaneously into C57BL/6 mice. Growth curves **(A)** and individual weights represented as scatter dot plot **(B)** of generated tumors. Results are shown as mean ± SEM (* p<0.01; n = 7 per group). **(C)** Representative photograph of the excised tumors. **(D)** Flow cytometric analysis of murine cell populations in B16-derived tumors obtained with the indicated shMyD88 and SCR cells. Representative dot plots showing percentages of vascular endothelial cells (CD31+) and myeloid subsets (within CD45+ gate). **(E)** Quantification of intra-tumor composition of indicated cell populations. Data presented as mean ± SEM (* p<0.01).

Based on the functional differences observed between SCR and shMyD88 cell lines, we further investigated whether MyD88 knockdown affected tumorigenicity *in vivo* by using a mouse tumor model. Stable B16 cells were subcutaneously injected into syngeneic C57BL/6 mice and monitored for melanoma development. We found that MyD88-downregulated B16 cells exhibited significantly impaired tumor growth compared with SCR control cells or non-transduced B16 cells (Fig 5A and 5B). We also observed a marked reduction in the final size and weight of the tumors formed from shMyD88 cells compared to those derived from SCR or non-transduced B16 cells (Fig 5B and 5C). To broaden this study, we included an analysis of the cellular composition of the tumor beds assessed by fluorescence-activated cell sorting. To evaluate the degree of tumor angiogenesis we determine the percentage of cells positive for the vascular marker CD31 among the total events acquired by flow cytometry (Fig C in S1 Fig). As shown in Fig 5D and 5E, reduced proportions of CD31+ cells were present in tumors obtained from shMyD88-injected mice, consistent with its decreased ability to induce angiogenesis. In addition, changes in the endothelial cell number coincided with changes in the immune cell composition. Whereas the number of tumor-infiltrating leukocytes (CD45+) was comparable between the groups, melanomas induced by MyD88 knockdown cells showed a myeloid infiltrate enriched predominantly in CD11b+F4/80+ macrophages and with a lesser percentage of CD11b+Gr1+ cells (Fig 5D and 5E). Again, tumors induced by SCR and non-transduced B16 cells show similar frequencies of infiltrating CD45+ cells as well as comparable proportions of the different myeloid population and CD31+ and Tie2+ cells (Fig 1B, Figs A and B in S1 Fig). Taken together, these findings offer evidence that MyD88 signaling effectively promotes angiogenesis and tumor growth *in vivo*.

## Discussion

The way molecular alterations in tumor cells affect tumor microenvironment and consequently tumor growth *in vivo* has only recently started to be studied. Interestingly, it has been reported that molecular changes in tumor cells that had no apparent impact on their proliferation capacity *in vitro*, had unexpected consequences in the tumor microenvironment *in vivo*. Indeed, when kinases of the Hippo pathway were inactivated in tumor cell lines, their anchorage-independent growth potential *in vitro* was increased but, surprisingly, this inactivation led to a profound inhibition of tumor growth *in vivo* due to changes in the antitumor immune response [29]. In this work, we demonstrate that silencing MyD88 in B16 cells, do not alter their proliferation/survival capacity *in vitro* but it inhibits tumor growth *in vivo*. This inhibition seems to be dependent on changes in the balance of chemokines and pro- and anti-angiogenic factors secreted by shMyD88 cells, which in turn elicits a different type of immune infiltration.

The mechanisms that link inflammatory responses to cancer development remain a subject of intense investigation, emphasizing the need to better understand the cellular and molecular pathways that create a tumor promoting microenvironment. Genetic alterations that cause cellular transformation can also drive the activation of intrinsic inflammation-related programs that leads to the expression of bioactive molecules that facilitate proliferation, angiogenesis, tumor invasion, and metastasis [30]. In this regard, the study of MyD88 adaptor protein and its role in tumorigenesis has gain great interest since it is placed at the crossroad of several signaling routes connecting innate receptors and pro-inflammatory mediators. A clear prerequisite for a tumor to grow is to acquire the ability to stimulate neovascularization by shifting the balance between positive and negative angiogenic regulators. Fast growing malignant cells produce cytokines and enzymes that induce the proliferation and migration of surrounding endothelial cells allowing blood vessel growth to ensure sufficient supply of oxygen and nutrients. Herein, we have demonstrated that MyD88 expression is required to maintain the angiogenic switch that supports B16 melanoma growth. By knocking down MyD88 we reduced TLR-mediated NF-κB activation with no evident effects over cell proliferation and survival. In addition, MyD88 downregulation was associated with a decrease in the levels of HIF1α and its target gene VEGF, in correlation with an impaired capability to induce capillary sprouting and tube formation of endothelial cells.

Some recent works have uncovered cell-autonomous functions of MyD88 relevant to oncogenesis and tumor progression. For instance, the TLR2/MyD88 axis has been recognized as a critical regulator of self-renewal of mammary and intestine epithelium as well as breast and colon cancer tumorigenesis [31]. Also, it was reported that repressing MyD88 in colon cancer cells leads to accumulation of DNA damage resulting in apoptosis dependent on p53 [16]. Furthermore, MyD88 could maintain the activation of PI3K/AKT and Ras/ERK pathways, thus contributing to epithelial-mesenchymal transition and cellular transformation, respectively [13,32]. Interestingly, MyD88 mutations caused constitutive activation of NF-kB and STAT3, cooperating to the pathogenesis of B cell malignancies [33]. Our data imply that MyD88 acts intrinsically to promote a protumorigenic tissue microenvironment. Engagement of MyD88 in activated murine melanoma cells triggers an angiogenic program that rely on the secretion of trophic factors for vascular cells, including VEGF, FGF-2, and CXCL15. The actions of MyD88 in regulating the expression of these factors is likely to involve both direct and indirect mechanisms, mainly coordinating the interplay between NF-κB, HIF1α, and STAT3 transcription factors, which are well known for its roles in tumor development and inflammation [34].

Early studies have focused on the functions of MyD88 in immune cells, more specifically, on phagocytic and antigen-presenting cells. MyD88 signaling endows these cells with the paradoxical ability to exert both tumor-suppressing and tumor-promoting effects, capable of either eliciting a productive antitumor adaptive immunity or promoting a tumor-enhancing immunosuppressive response [34]. Here, we revealed that melanomas developed from cells lacking MyD88 have an improved infiltration of macrophages to the tumor site. This can be a consequence of the enhanced secretion of chemoattracting ligands involved in the recruitment of this cell linage by shMyD88 cells, including CCL2, CXCL10, and CXCL1 [35]. It has been previously reported that B16 melanomas are infiltrated with tumor associated macrophages skewed towards an alternative M2-polarized phenotype, which can promote angiogenesis and tissue remodeling, thereby supporting tumor progression and metastatic spread [35]. This effect, together with a shrinkage in the number of Gr1+ CD11b+ cells (that could be considered immature myeloid cells), might represent a compensatory response to hypoxic avascular tumors. Nonetheless, it has been established that an important mechanism involved in the inherent tumor refractoriness to angiogenic blockade by anti-VEGF therapy is the production of additional proangiogenic molecules besides VEGF by infiltrating myeloid cells, or the recruitment of proangiogenic bone marrow-derived cells [36,37].

Modulators of the TLR/MyD88 pathway are currently under investigation in clinical trials for cancer treatment. The use of TLR agonists in antineoplastic therapy is based on its ability to activate antigen-presenting cells which in turn, can orchestrate a tumor-specific immune response. TLR ligands are being tested as vaccine adjuvants together with tumor antigens or used in combination with chemotherapeutic drugs or radiotherapy to synergistically enhance their immunostimulatory capacity [38]. However, there is a potential for TLR signaling to occur on either the tumor vasculature or tumor cells themselves that could negate antitumor responses or, as we evidenced in our model, promote tumor growth. Moreover, persistent activation of signals triggered by DAMPs released by dying cells may promote carcinogenesis in a TLR-dependent manner [6]. A paradigmatic case could be HMGB1, a nuclear non-histone architectural protein involved in DNA binding that can be released after cellular stress and drive TLR4-dependent inflammatory responses [39,40]. The presence of cells with cytoplasmic HMGB1 was appreciated by immunofluorescence in sections from B16 tumors and this localization occurs in the context of active HMGB1 release which usually precedes necrosis [41]. Therefore, released HMGB1 could activate the MyD88 pathway in tumor cells and mediate amplification of angiogenesis which can be complementary to the direct effect of HMGB1 on endothelial cells to enhance the expression of proteins involved in proangiogenic cascades, thus contributing to tumor vascularization [42]. Consequently, targeting TLR4/MyD88 signaling may be an attractive therapeutic strategy to hinder tumor progression. Though, the final outcome will depend on the TLR expression profile, the specific cellular context, and the balance between the effects of concurrent inhibition of the pathway on malignant cells and innate immune cells.

In conclusion, our current study defines that downregulation of MyD88 in murine melanoma B16 cells suppresses its angiogenic potential, culminating in significantly impaired subcutaneous tumor growth, associated with a dysregulation of the expression of genes involved in inflammation and angiogenesis. Although extensive work, including especially the investigation of precise MyD88 functions in human melanoma cells, will be needed to elucidate the complete mechanism involved, our results suggest that inhibitors of the MyD88 pathway merit investigation as possible therapeutic and chemoprevention agents.

## Materials and methods

### Mice and tumor xenografts

C57BL/6 mice were obtained from Universidad Nacional de La Plata (Buenos Aires, Argentina). Mice were maintained under specific pathogen-free conditions at the Animal Resource Facility of the Clinical Biochemistry and Immunology Research Center (CIBICI -CONICET) and were monitored every other day by the animal house staff. Experiments were in compliance with the Guide for the Care and Use of Laboratory Animals published by the NIH and approved by the by the Comité de Ética de Protocolos Experimentales—Facultad de Ciencias Químicas—Universidad Nacional de Córdoba (Res HCD 1017/2015).

For tumor induction, 1×10^6^ B16 cells were administered s.c. in 200 μl of sterile saline into the right flank of 6-week-old male C57BL/6 mice. Animals were followed weekly to early identify tumour onset by manual palpation and tumor size was measured every 2–3 days with a caliper. Tumor volume was calculated as (D×d2)/2, where D and d refer to the long and short tumor diameter, respectively. The mice were euthanized by cervical dislocation after anesthetization with 4% chloral hydrate v/v (Sigma-Aldrich) when the tumors exceed a volume of 2 cm3 or when other signs of discomfort were observed.

### Reagents and cell lines

LPS from *Escherichia coli* 055:B5 and glycyrrhizin were purchased from Sigma-Aldrich. Disulfide HMGB1 (LPS-free) was obtained from HMGBiotech. A list with details of antibodies used can be found in S2 Table.

Murine B16-F0 melanoma cell line was previously described [43]. Cells were maintained in Dulbecco’s modified Eagle medium (DMEM, Gibco) supplemented with 10% heat-inactivated fetal bovine serum (Gibco) and 100 IU/mL penicillin/streptomycin (Gibco). The media for shRNA-expressing cells contained in addition 2 μg/ml puromycin (Sigma-Aldrich). All cells were grown in a humidified incubator at 37°C with 5% CO_2_ and were negative for mycoplasma contamination tested regularly by PCR.

### Lentiviral vectors and knockdown

Generation of lentiviruses encoding shRNAs against MyD88 was performed as previously described [44]. The 21-mer target sequences were scrambled (SCR), 5’-GTTAACTGCGTACCTTGAGTA; shMyD88-A, 5’-GCGACTGATTCCTATTAAATA; and shMyD88-B, 5’- GCCAGCGAGCTAATTGAGAAA. HEK293T cells were used for preparation of lentiviral stocks. Cells were cotransfected with the two helper plasmids (pCMV-dR8.2 and pVSV-G) along with shRNA-pLKO.1 vector, using Lipofectamine 2000 reagent (Invitrogen). Supernatants containing infectious viral particles were harvested 48–72 h post-transfection and filtered. Infections of B16 cells were performed with virus-containing supernatant supplemented with 6 μg/mL polybrene (Sigma). Stable cell lines were selected with 2 μg/ml of puromycin (Sigma-Aldrich) for 2 weeks.

### Quantitative real time RT-PCR

Total RNA was extracted from cultured cells using Trizol Reagent (Life Technologies), and reverse-transcribed using random primers (Promega) and M-MLV reverse transcriptase (Promega). Specific transcripts were quantified by real time qRT-PCR (StepOnePlus Real-Time PCR System, Applied Biosystems) using the StepOne Software v2.2.2. Experiments were performed using SYBR Green PCR Master Mix (Applied Biosystems). Primer sequences are indicated in S1 Table. The cycling conditions included a hot start at 95°C for 10 min, followed by 40 cycles at 95°C for 15 s and 60°C for 1 min. Specificity was verified by melting curve analysis and agarose gel electrophoresis. Fold change in gene expression was calculated according to the 2^-ΔΔCt^ method. Each sample was analyzed in triplicate. No amplification was observed in PCR reactions containing water or RNA samples incubated without reverse transcriptase during cDNA synthesis as template.

### In Cell Western assays

Cells were seeded in 96-well plates at 3×10^4^ cells/well. After treatment, cells were washed twice with PBS, fixed with 4% paraforma1dehyde for 20 min, and permeabilized in 0.01% Triton X-100. Blocking was carried out with Odyssey blocking buffer (LICOR Bioscience). Each well was incubated overnight at 4°C with corresponding target primary rabbit antibody (see S2 Table) and mouse anti-α-tubulin as control, followed by IRDye conjugated anti-rabbit (800 nm) and anti-mouse (700 nm) secondary antibodies incubation at room temperature for 1 h in the dark. A row of cells was included on every plate for incubation with secondary antibodies alone to determine any background level of labelling. Plates were imaged on an Odyssey Infrared Scanner (LI-COR Biosciences) and data analyzed using Image Studio 3.1 software.

### Cell proliferation and viability

Proliferation rate and cell survival were evaluated with the CellTiter 96 AQueous Non-Radioactive Cell Proliferation Assay kit (Promega). In brief, for cell proliferation, B16 cells were plated in triplicate wells of a 96-well plate at a density of 3×10^3^ cells/well and further treated as indicated. In the case of viability assays, 1×10^4^ cells/well were seeded and incubated with media containing dimethyl sulfoxide (vehicle, Sigma-Aldrich), doxorubicin (Sigma-Aldrich), or cis-diammineplatinum (II) (Sigma-Aldrich). After 48 h, cell densities were measured following incubation for 2 h with a combined MTS/PMS solution and absorbance was measured at 490 nm on a microplate reader (Bio-Rad). Cellular growth was calculated from the absorbance ratio between values obtained at the end point with respect to those measured in cells before treatment. All assays were independently performed three times.

### Cell cycle analysis

Exponentially growing B16 cell lines were treated with vehicle or LPS as indicated. After the harvest, cell cycle analysis was performed as it was described before [44].

### Reporter assay

The NF-κB reporter plasmid used for promoter activity assays has been already described [45]. B16 cells were seeded at a density of 1×10^5^ per well in 24-well plates, cultured for 24 h and afterwards transfected with 1 μl of Lipofectamine 2000 reagent (Invitrogen), 500 ng of the reporter construct and 50 ng of the Renilla normalizing vector. As controls, cells were transfected with a CMV promoter-driven luciferase vector (pCMV-Luc). Luciferase and Renilla activities were measured on a GloMax-Multi Detection System (Promega) using the Dual-Luciferase Reporter Assay System (Promega), according to the manufacturer's instructions. Luciferase activity was normalized by the values of the Renilla luciferase and expressed as relative light units.

### Antibody array

For the cytokine array, 1×10^6^ cells were plated onto 100 mm plates in tissue culture media and grown to 90% confluence. Media was replaced with 5 ml of fresh DMEM supplemented with 1% serum 24 h prior the experiment. Culture supernatants were harvested and diluted according to the final cell number. The expression of angiogenesis-related proteins was detected using the Proteome Profiler Mouse Angiogenesis Array Kit (ARY015; R&D Systems) following the manufacturer’s protocols. Developed films were scanned and analyzed by quantifying the mean spot pixel densities using the NIH ImageJ software. Averaged background signal was subtracted and corresponding signals on different arrays were normalized using internal controls.

### In vitro capillary tube formation assays

HMEC-1 were seeded on growth factor-reduced matrigel-coated plates (BD Biosciences) at a density of 1.5×10^4^ cells/well. Culture medium was replaced by supernatants from each B16 cell line and endothelial responses were assessed after 18 h. Tube formation was examined under an inverted light microscope, and the total number of branch points was determined with ImageJ software. Experiments were performed independently at least twice. Representative experiments are shown.

### Flow cytometry

The analysis of tumor-infiltrating cells was performed as described previously [46]. Briefly, mice were euthanized and whole tumors surgically resected, minced into smaller sections, and mechanically dissociated. Single-cell suspensions were prepared by enzymatic digestion and submitted to Ficoll-Hypaque (GE Healthcare) gradient centrifugation. Cells were subsequently washed with PBS containing 0.1% serum and stained with antibodies to detect endothelial and immune cells (see S2 Table) diluted in FACS buffer (EDTA 5 mmol/L, sodium azide 0.1%, BSA 1% in PBS). Analysis was conducted in a FACS Canto II flow cytometer (BD Biosciences) using FlowJo software (Tree Star). To evaluate the number of cells expressing the endothelial markers CD31 and Tie2, tumors were dissociated as described below and then a fixed number of cells were acquired in a storage gate that was exactly the same for all the samples analyzed. Results are expressed as % of total events.

### Immunofluorescence

Tumors for immunofluorescence staining were obtained after overnight fixation with 4% buffered paraformaldehyde. Then, they were dehydrated with 30% sucrose in PBS for 72 h, embedded in OCT compound, and stored at -80°C. Frozen tumors were sectioned into 14-mm-thick sections with a Leica cryostat. Slices were permeabilized in 0.01% Triton X-100, blocked in 0.4% fish skin gelatin and 0.2% Tween-20, and incubated with anti-HMGB1 in PBS-2.5% goat serum. Secondary staining proceeded with the anti-rabbit Alexa Fluor 488-conjugated antibody in PBS-2.5% goat serum. Nuclei were counterstained with DAPI. Slides were mounted with FluorSave Reagent (Calbiochem), and images were acquired using a Leica DMi8 microscope (Leica Microsystems). Images were processed with Adobe Photoshop CC software.

### Western blot

Culture supernatants were concentrated using the Vivaspin sample concentrator (GE Healthcare Life Science) and prepared in Laemmli buffer (60 mM Tris-HCl pH 6.8, 10% glycerol, 2% sodium dodecyl sulphate, 1% 2-b-mercaptoethanol, 50 mM DTT, 0.01% bromophenol blue). Proteins were resolved on 10% SDS-PAGE gels and transferred to nitrocellulose membranes (GE Healthcare) according to standard techniques. The membranes were incubated with primary antibody against at 4°C overnight. Detection of specific bands was achieved using fluorescent-labeled secondary antibody and imaged using the LI-COR Odyssey system.

### Statistical analysis

Data were analyzed by one-way analysis of variance (ANOVA) followed by Dunnett’s multiple comparison *post hoc* test using GraphPad Prism version 6.0 (GraphPad Software). Comparisons between two groups were made by Student’s t test. Unless specified otherwise, all data are presented as means ± sem, and a *p* value < 0.05 was considered significant.

## Supporting information

S1 Fig Comparable cellular composition of the tumor beds induced by non-transduced and SCR transduced B6 cells. (A) Flow cytometric analysis of murine cell populations in B16-derived tumors obtained with the indicated non-transduced and SCR transduced B16 cells. Left panel: Representative dot plots showing percentages of vascular endothelial cells (CD31+ and Tie 2+ cells) and tumor infiltrating leucocytes (CD45+ cells). Right panel: Distribution of myeloid cell population among the tumor infiltrating CD45+ cells: CD11b+ F480+ (macrophages) and CD11b+ and Ly6C+ monocytes).B) Number of vascular endothelial cells (CD31+ and Tie 2+ cells) and myeloid cells (CD11b+ F480+ and CD11b+ Ly6C+) per gram of tumors derived from non-transduced and SCR transduced B16 cells. (B) Gate strategy used to analyze the frequency of CD31+ cells among the total number of cells present in a tumor homogenate. A fixed number of cells were acquired in a storage gate that was the same for all the samples analyzed. Results are expressed as % of total events.
(TIF)

S1 Table Primer sequences used for real time-PCR. (PDF)

S2 Table Antibodies. (PDF)
