# Supplementary figures and images for: Downregulation of adaptor protein MyD88 compromises the angiogenic potential of B16 murine melanoma
Source: PLoS One. 2017 Jun 29;12(6):e0179897. doi: 10.1371/journal.pone.0179897 (PMC5491060; doi:10.1371/journal.pone.0179897)

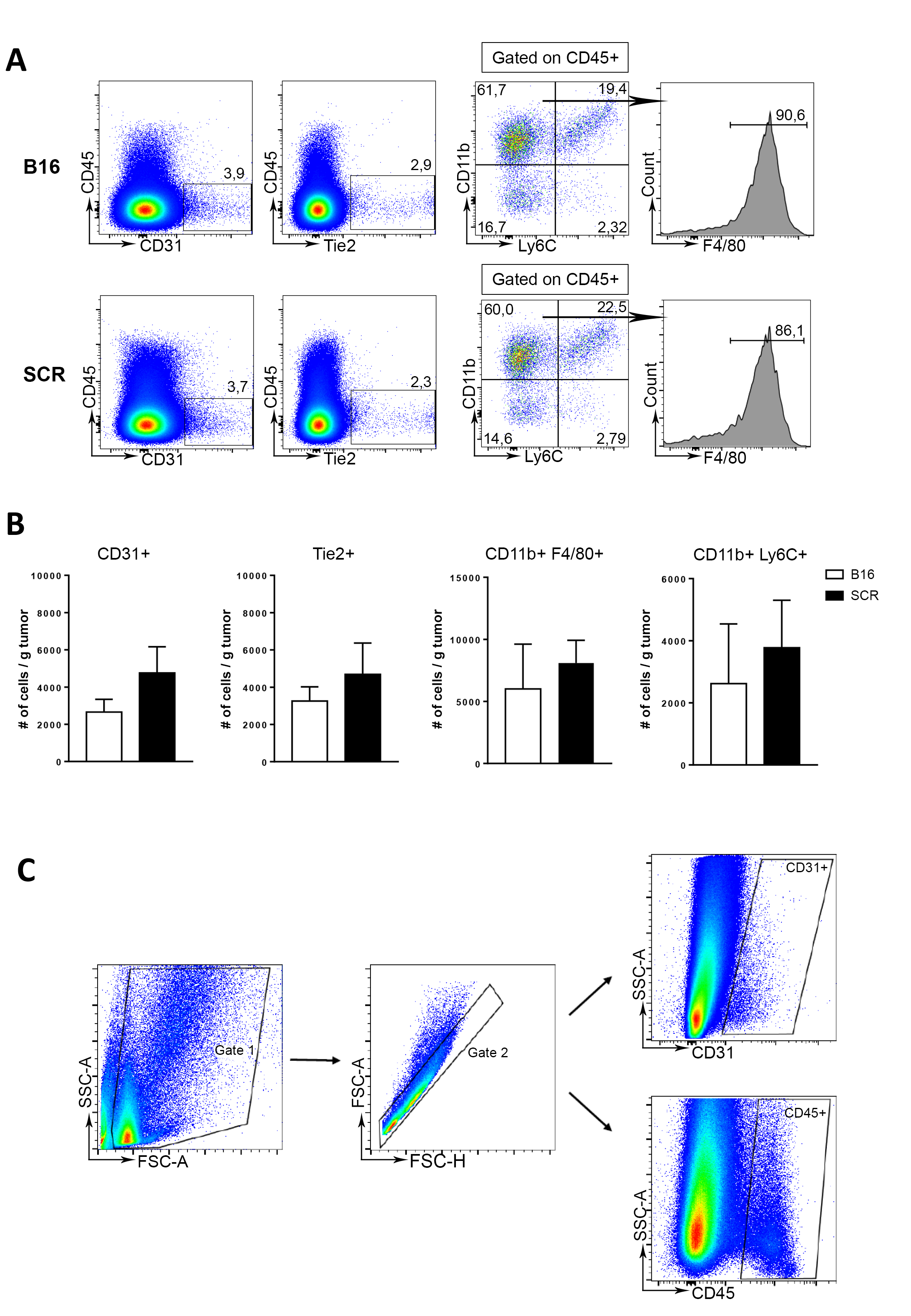

Supplement: S1 Fig — (A) Flow cytometric analysis of murine cell populations in B16-derived tumors obtained with the indicated non-transduced and SCR transduced B16 cells. Left panel: Representative dot plots showing percentages of vascular endothelial cells (CD31+ and Tie 2+ cells) and tumor infiltrating leucocytes (CD45+ cells). Right panel: Distribution of myeloid cell population among the tumor infiltrating CD45+ cells: CD11b+ F480+ (macrophages) and CD11b+ and Ly6C+ monocytes).B) Number of vascular endothelial cells (CD31+ and Tie 2+ cells) and myeloid cells (CD11b+ F480+ and CD11b+ Ly6C+) per gram of tumors derived from non-transduced and SCR transduced B16 cells. (B) Gate strategy used to analyze the frequency of CD31+ cells among the total number of cells present in a tumor homogenate. A fixed number of cells were acquired in a storage gate that was the same for all the samples analyzed. Results are expressed as % of total events. (TIF) [file pone.0179897.s001.tif]
