# Supplementary material for: Downregulation of adaptor protein MyD88 compromises the angiogenic potential of B16 murine melanoma
Source: PLoS One. 2017 Jun 29;12(6):e0179897. doi: 10.1371/journal.pone.0179897 (PMC5491060; doi:10.1371/journal.pone.0179897)
Supplement: S1 Table — (PDF) [file pone.0179897.s002.pdf]

S1 Table.Primer sequences used for Real Time-PCR

Gene	GeneBank ID	Forward primer (5'-3')	Reverse primer (5'-3')
Tgfb1	NM_011577.2	TGACGTCACTGGAGTTGTACGG	GGTTCATGTCATGGATGGTG
Il6	NM_031168.2	TCTGCAAGAGACTTCCATCCAGT	GGCAAATTTCCTGATTATATCC
Ifnb1	NM_010510.1	TTACACTGCCTTTGCCATCC	ACTGTCTGCTGGTGGAGTTCAT
Myd88	NM_010851.2	CATGGTGGTGGTTGTTTCTGAC	TGGAGACAGGCTGAGTGCAA
Hif1a	NM_001313919.1	ACCTTCATCGGAAACTCCAAAG	CTGTTAGGCTGGGAAAAGTTAGG
Vegfa	NM_001025250.3	CTTGTTCAGAGCGGAGAAAGC	ACATCTGCAAGTACGTTCGTT
Il8	NM_011339	GATATTCGAGACCATTTACTGCAAC	CTTCATTGCCGGTGGAAATTC
