# Supplementary material for: Downregulation of adaptor protein MyD88 compromises the angiogenic potential of B16 murine melanoma
Source: PLoS One. 2017 Jun 29;12(6):e0179897. doi: 10.1371/journal.pone.0179897 (PMC5491060; doi:10.1371/journal.pone.0179897)
Supplement: S2 Table — (PDF) [file pone.0179897.s003.pdf]

S2 Table.Antibodies

Target protein	Antibody name	Source	Cat. #	Applications
**MyD88**	MyD88 (D80F5) Rabbit mAb	Cell Signaling Technology	4283	ICW
**p-STAT3**	Phospho-Stat3 (Tyr705) (D3A7) XP® Rabbit mAb	Cell Signaling Technology	9145	ICW
**p-ERK**	Phospho-p44/42 MAPK (Thr202/Tyr204) (D13.14.4E)	Cell Signaling Technology	4370	ICW
**α-tubulin**	Monoclonal Anti-α-Tubulin	Sigma-Aldrich	T9026	ICW
**HIF1α**	HIF-1α Antibody (H-206)	Santa Cruz Biotechnology	sc-10790	ICW
**CD31**	FITC Rat Anti-Mouse CD31	BD Pharmingen	553372	FC
**CD45**	APC/Cy7 anti-mouse CD45 Antibody	BioLegend	103115	FC
**F4/80**	Alexa Fluor® 647 anti-mouse F4/80 Antibody	BioLegend	123121	FC
**Tie2 (CD202b)**	PE anti-mouse CD202b Antibody	BioLegend	124007	FC
**Ly6C**	APC anti-mouse Ly-6C Antibody	BioLegend	128016	FC
**Gr1**	PE Rat Anti-Mouse Ly-6G and Ly-6C	BD Pharmingen	553128	FC
**CD11b**	FITC anti-mouse/human CD11b Antibody	BioLegend	101205	FC
**HMGB1**	Anti-HMGB1 antibody [EPR3507]	Abcam	ab79823	IF, WB
**Anti-rabbit IRDye 800**	IRDye® 800CW Goat anti-Rabbit IgG	LI-COR Biosciences	926-32211	ICW
**Anti-mouse IRDye 680**	IRDye® 680RD Goat anti-Mouse IgG	LI-COR Biosciences	926-68070	ICW
**Anti-rabbit Alexa 488**	Goat Anti-Rabbit Alexa Fluor® 488	Molecular Probes	A-11034	IF
